# Genetic polymorphisms and gene-dosage effect in ovarian cancer risk and response to paclitaxel/cisplatin chemotherapy

**DOI:** 10.1186/s13046-015-0124-y

**Published:** 2015-01-16

**Authors:** Karolina Tecza, Jolanta Pamula-Pilat, Zofia Kolosza, Natalia Radlak, Ewa Grzybowska

**Affiliations:** Center for Translational Research and Molecular Biology of Cancer, Maria Sklodowska-Curie Memorial Cancer Center and Institute of Oncology, Gliwice Branch, Wybrzeze Armii Krajowej 15, 44-101 Gliwice, Poland; Department of Epidemiology and Silesia Cancer Registry, Maria Sklodowska-Curie Memorial Cancer Center and Institute of Oncology, Gliwice Branch, Gliwice, Poland; Institute of Automatic Control, Silesian University of Technology, Gliwice, Poland

**Keywords:** Ovarian cancer, Pharmacogenetics, Cancer susceptibility, Clinical course, Treatment outcome

## Abstract

**Background:**

Ovarian malignancies are often diagnosed in advanced stage and at the same time resistance to treatment, both intrinsic and developed during treatment, is sometimes observed. These facts underscore the need for new markers of ovarian cancer risk, as well as markers of treatment effectiveness.

**Methods:**

In this study we genotyped 225 ovarian cancer patients, 64 breast and ovarian cancer patients and 348 healthy controls. In total, 12 polymorphic variants and 2 deletions in *PGR, ABCB1, ABCG2, GSTT1, GSTM1, GSTP1, ATM, TP53* and *ATP7B* genes were analyzed using ASA-PCR, RFLP-PCR, multiplex-PCR and sequencing.

**Results:**

Ten genetic polymorphisms were significantly associated with the risk of developing ovarian carcinoma in at least one of the groups under study. Impact of *PGR* gene polymorphisms on ovarian cancer risk was specific only for the group of the *BRCA1* mutation carriers (in presence of p.Val660Leu variant- OR 2,82; p = 0,010), which confirms the difference in modulation of ovarian cancer risk between sporadic and hereditary malignancies, including the breast-ovarian cancer group (as a cancer-prone group). The analyses showed also the importance of *ATP7B* gene in ovarian carcinogenesis, both studied variants of which significantly modulated the ovarian cancer risk in all groups excluding the group with *BRCA1* mutation. Cumulative risk analysis revealed 3 unfavorable variants that increased significantly the risk of developing ovarian cancer (p.Ile1145 = *ABCB1*+ p.Asp1853Asn *ATM*+ p.Ser406Ala *ATP7B*- OR 7,47; p = 0,002) and significantly modified the progression free survival (PFS) of the patients, and also two favorable genotypes which protected against ovarian cancer (p.Arg952Lys *ATP7*B+ p.Arg72Pro TP53- OR 0,50; p = 0,008). PFS analysis for carriers of favorable versus unfavorable genotypes emphasized the impact of the regulation of cell cycle (p.Asp1853Asn *ATM*) and active transport of xenobiotics (p.Ser894Ala/Thr *ABCB1*) on the risk of disease progression (HR 3,81; p = 0,010) after paclitaxel/cisplatin chemotherapy.

**Conclusions:**

The unfavorable genetic variants could facilitate carcinogenic process and once their carriers developed malignancy, their chances of survival were smaller. Our analyses also showed a strong gene-dosage effect with the decrease of progression-free survival for the carriers of two unfavorable genetic factors.

## Background

Ovarian carcinoma is the sixth leading malignancy diagnosed in women and fourth leading cause of cancer mortality in Silesia, a southern province of Poland [1]. Poland belongs to the countries with high morbidity rates for ovarian carcinoma - epidemiologic data show steady rise of ovarian carcinoma incidence [2]. Mortality rates differ for different continents and geographic regions, countries, races and ethnic groups. Malignancies of the ovary are diagnosed frequently in advanced stage in postmenopausal women, contributing to a relatively high mortality and little progress in improving survival rates within the past few decades [3,4]. These facts emphasize the necessity of developing better methods of treatment, diagnosis and prevention. Unfavorable statistics in ovarian cancer patients reflects in part poor understanding of molecular pathogenesis and progression of the disease [5].

The strongest risk factor of developing ovarian cancer is the family history of breast and ovarian cancer. About 15% of ovarian cancer patients in Polish population carry germline mutation in *BRCA1/2* genes [6]. A small number of patients with inherited predisposition to ovarian carcinoma is also connected with Lynch syndrome and germline mutations in mismatch repair genes, such as *hMLH1, hMSH2, hMSH6, PMS1* and *PMS2* [7,8]. However, the common genetic polymorphisms can also influence the risk of developing ovarian carcinoma.

In this study we wanted to evaluate the effects of polymorphic variants of the genes connected with progesterone activity, cell cycle control, and transport systems on ovarian cancer risk. Besides frequent occurrence of germline mutations in *BRCA1/2* genes, ovarian carcinoma is sometimes accompanied by breast carcinoma, so we divided the group of ovarian cancer patients under study into four groups: all patients, patients without *BRCA1* mutation, patients with germline mutation in *BRCA1* gene and patients who developed both breast and ovarian carcinoma. The aim was to find out whether there were differences in cancer risk among these groups.

## Methods

### Patients and controls

A total of 225 case subjects diagnosed with ovarian cancer and 64 with breast and ovarian cancer participated in the analysis. Cases of ovarian malignancies other than epithelial, i.e. germ cell and sex cord stromal tumors, were not included in this study. Patients filled an informed consent form and agreed to have their genetic material used for research purpose. The majority of patients (186 patients, 82.6%) were diagnosed between year 2000 and 2006, while 33 women (14.7%) had been diagnosed during the 1983–1999 time period. Exact diagnosis date was not known for 6 (2.7%) patients. Anonymous healthy women (n = 348) forming the control group were recruited from among female employees of the Cancer Center and Institute of Oncology in Gliwice and were matched to the study groups for mean age of ovarian cancer diagnosis. The study was approved by the appropriate bioethics committee. Status of the most common *BRCA1* mutations in Silesian population was determined for all subjects under study, including the control group. None of control subjects carried germline mutation in *BRCA1* gene. For statistical analysis, study groups were divided with respect to the absence of germline mutation (*BRCA1*-) or its presence (*BRCA1*+), or were analyzed as a total group. For some analyses the calculations were done for *BRCA1*- group only as *BRCA1*+ group was much smaller and some calculations were not possible. The group of breast and ovarian cancer cases was analyzed separately, without *BRCA1* mutation-based division due to small number of subjects. All women in the studied groups were Caucasian with Silesian descent. The observation ended on 1st of February 2011. Full clinical characteristics of the patient groups are presented in Table 1.

**Table 1:** **Clinical characteristics of patient groups**

	**Ovarian cancer group (n = 225)**	**Breast and ovarian cancer group (n = 64)**
Mean age at ovarian cancer diagnosis in years (range)	48.8 (16.9-70.6)	51.2 (33.8-66.9)
Mean age at breast cancer diagnosis in years (range)		48.1 (25.0-72.9)
*BRCA1* mutation status		
• Negative	198 (88.0%)	46 (71.9%)
• Positive	27 (12.0%)	18 (28.1%)
*BRCA1* mutation type		
• 5382insC (c.5266dupC)	17 (63.0%)	14 (77.8%)
• 300 T/G (c.181 T > G)	8 (29.6%)	2 (11.1%)
• 185delAG (c.68-69delAG)	2 (7.4%)	2 (11.1%)
• 4153delA (c. 4034delA)	--	--
• 3819del5 (c.3700_3704del5)	--	--
Ovarian Cancer		
FIGO		
• I	73 (32.4%)	16 (25.0%)
• II	33 (14.7%)	5 (7.8%)
• III	95 (42.2%)	31 (48.5%)
• IV	13 (5.8%)	2 (3.1%)
• Unspecified/unknown	11 (4.9%)	10 (15.6%)
GRADE		
• G1- well differentiated	34 (15.1%)	3 (4.7%)
• G2- moderately differentiated	98 (43.6%)	20 (31.2%)
• G3- undifferentiated	86 (38.2%)	29 (45.3%)
• G4	1 (0.4%)	--
• unspecified/unknown	6 (2.7)	12 (18.8%)
Histology		
• Serous	121 (53.8%)	41 (64.1%)
• Mucinous	23 (10.2%)	2 (3.1%)
• Endometrioid	33 (14.7%)	2 (3.1%)
• Clear cell	6 (2.7%)	1 (1.6%)
• Other/unspecified	41 (18.2%)	15 (23.4%)
• Unknown	1 (0.4%)	3 (4.7%)
Breast Cancer		
GRADE		
• G1- well differentiated		10 (15.6%)
• G2- moderately differentiated		22 (34.4%)
• G3- undifferentiated		6 (9.4%)
• Unspecified/unknown		26 (40.6%)
Histology		
• Ductal carcinoma *in situ*		1 (1.6%)
• Ductal carcinoma		44 (68.7%)
• Lobular carcinoma		1 (1.6%)
• Medullary carcinoma		3 (4.7%)
• Other		13 (20.3%)
• Unspecified		2 (3.1%)

For survival analysis, 129 ovarian cancer patients diagnosed between 2000 and 2006 were selected. These women underwent primary cytoreduction prior to paclitaxel/cisplatin first-line chemotherapy. The surgeries were performed in other hospitals, therefore the data regarding residual disease were not available. Majority of the selected patients (119 women; 92.2%) were given six courses of first-line treatment, one patient underwent eight cycles while nine patients obtained less than six cycles, due to severe adverse reaction or progression during treatment, or because of complete response. Treatment response according to RECIST (version 1.0) was determined after the completion of chemotherapy. Complete response (CR) was achieved in 97 patients (75.2%), whereas 15 subjects (11.6%) showed partial response (PR), and four patients (3.1%) progressed (PD) during treatment. Disease stabilization (SD) was not observed in selected group of patients. The treatment response estimation was not available for 13 women (10.1%).

### Clinical endpoints

Overall survival (OS) was calculated as time period (in months) from diagnosis (established as the surgery date) to death from any cause, or to date of last contact with the patient. Median overall survival was 86.8 months; during the observation 58 patients (45.0%) from the paclitaxel/cisplatin group died. Progression-free survival (PFS) was calculated as time period (in months) from date of first course of chemotherapy to date of progression (confirmed by the MRI, CT or ultrasound), or to date of last contact with the patient. Disease progression was defined as presence of distant metastasis, the local recurrences were not analyzed. Progression was confirmed in 38 patients (29.5%), while 88 (68.2%) women had no metastases till the end of observation. Three patients (2.3%) were rejected from PFS analysis due to lack of data.

### Genes and polymorphism selection

Genes selected for this study represent three functional groups important in ovarian cancer development and patients’ prognosis, i.e. hormonal control (*PGR-* progesterone receptor), xenobiotics removal (*ABCB1-* ATP-binding cassette subfamily B, member 1; *ABCG2*- ATP-binding cassette sub-family G, member 2; *ATP7B*- ATPase, Cu++ transporting, beta polypeptide; *GSTT1*- glutathione S-transferase theta 1; *GSTM1*- glutathione S-transferase mu 1; *GSTP1*- glutathione S-transferase pi 1) and DNA damage repair (*ATM*- ATM serine/threonine kinase; *TP53*- tumor protein p53). The selection of polymorphisms was based on literature search as well as search in the public databases (NCBI dbSNP, PharmGKB and HapMap). All selected variants had MAF higher than 5% in the European Caucasian population.

### Genotyping

Genomic DNA was isolated from the peripheral blood leukocytes using the phenol-chloroform method. Genotyping was performed using the allele-specific amplification PCR (ASA-PCR), multiplex-PCR or RFLP-PCR method. Genotyping of polymorphic variants in *PGR* (rs10895068 and p.Val660Leu), *ABCB1* (p.Ile1145 = and p.Ser893Ala/Trp), *ABCG2* (p.Gln141Lys), *ATM* (p.Asp1853Asn), *TP53* (p.Arg72Pro), *GSTP1* (p.Ile105Val) genes, as well as detection of *GSTT1*/*M1* gene deletions, were performed as described previously [9-17]. The genotyping methods for polymorphisms in *ATP7B* (p.Ser406Ala and p.Arg952Lys), *ABCG2* (rs13120400) and *PGR* gene (rs474320) were developed for this study.

### Statistical analysis

The difference between observed and expected genotype frequencies in control and patient groups were tested for Hardy-Weinberg Equilibrium (HWE) using the *χ*^2^ test. Statistical significance threshold was set at 0.05; all results with p-value ≤0.1 were seen as trend indicators. In all analyses, common homozygotes were used as a reference genotype with respect to heterozygotes, rare homozygotes and rare allele carriers. In case of trinucleotide polymorphism rs2032582 in *ABCB1* gene, an additional combination was applied, comparing the carriers of common G allele with the carriers of rare alleles (i.e. T and A). In case–control analysis, odds ratios (ORs), 95% confidence intervals (95% CIs) and p values were set to test associations between genotype distributions in cases and their controls, and logistic regression model was applied. Survival curves were derived by Kaplan-Meyer method, p values were computed by log-rank test. The relative risk of death and progression was estimated as hazard ratios (HRs); 95% confidence intervals (95% CIs) and p value were determined by Cox proportional hazard regression model. SNPs correlated with survival were re-evaluated in the multivariate analyses adjusted to known ovarian cancer prognostic factors (cancer histotype and FIGO stage). Age at time of diagnosis, tumor GRADE and the presence of *BRCA1* mutation were not associated with patients’ survival and these factors were not included in multivariate model adjustment. All statistical calculations were performed using Statistica v.7.0 software (StatSoft).

## Results

### Case–control analyses

The SNPs under study did not show significant departure from Hardy-Weinberg equilibrium with the exception of GSTP1 p.Ile105Val polymorphism which significantly departed from Hardy-Weinberg equilibrium (p = 0.032) in the control group (data not shown). Among twelve polymorphic variants and two deletions in nine genes under study we found ten which were significantly associated with the increased or decreased risk of developing ovarian carcinoma in at least one of the groups under study (Table 2).

**Table 2:** **Case–control analyses of ovarian and breast and ovarian cancer risk**

			**Ovarian cancer all patients**			**Ovarian cancer** ***BRCA1*** **-**			**Ovarian cancer** ***BRCA1*** **+**			**Breast and ovarian cancer**		
**Gene polymorphism**	**Genotype**	**Controls n(%)**	**n(%)**	**OR (±95% CI)**	**p**	**n(%)**	**OR (±95% CI)**	**p**	**n(%)**	**OR (±95% CI)**	**p**	**n(%)**	**OR (±95% CI)**	**p**
*PGR* p.Val660Leu rs1042838	GG	239 (69.3)	143 (63.9)	1(ref)		131 (66.5)	1(ref)		12 (44.4)	1(ref)		46 (75.4)	1 (ref)	
	GT	95 (27.5)	74 (33.0)	1.03 (0.90-1.88)	0.160	60 (30.5)	1.15 (0.78-1.70)	0.473	14 (51.9)	**2.94** (1.31-6.58)	**0.009**	14 (23.0)	0.77 (0.40-1.46)	0.416
	TT	11 (3.2)	7 (3.1)	1.06 (0.40-2.81)	0.901	6 (3.0)	1.00 (0.36-2.75)	0.993	1 (3.70)	1.81 (0.22-15.20)	0.584	1 (1.6)	0.47 (0.06-3.75)	0.478
	GT + TT	106 (30.7)	81 (36.1)	1.20 (0.88-1.63)	0.249	66 (33.5)	1.14 (0.78-1.65)	0.504	15 (55.6)	**2.82** (1.28-6.23)	**0.010**	15 (24.6)	0.74 (0.39-1.38)	0.336
*ABCB1* p.Ser893Ala p.Ser893Thr rs2032582	GG	117 (33.7)	65 (29.0)	1(ref)		56 (28.4)	1(ref)		9 (33.4)	1(ref)		16 (26.2)	1 (ref)	
	GT	156 (45.0)	115 (51.4)	1.33 (0.90-1.95)	0.152	104 (52.8)	1.39 (0.93-2.09)	0.108	11 (40.7)	0.92 (0.37-2.28)	0.852	33 (54.1)	1.55 (0.71-2.94)	0.184
	TT	60 (17.3)	37 (16.5)	1.11 (0.67-1.85)	0.688	32 (16.3)	1.11 (0.65-1.90)	0.691	5 (18.5)	1.08 (0.35-3.38)	0.890	9 (14.8)	1.10 (0.46-2.63)	0.836
	GA	9 (2.6)	2 (0.9)	0.40 (0.08-1.91)	0.250	2 (1.0)	0.46 (0.10-2.24)	0.337	--	--	--	2 (3.3)	1.63 (0.32-8.31)	0.557
	TA	5 (1.4)	5 (2.2)	1.80 (0.50-6.45)	0.367	3 (1.5)	1.25 (0.29-5.49)	0.763	2 (7.4)	**5.20** (0.87-31.18)	**0.069**	1 (1.6)	1.46 (0.16-13.6)	0.736
	AA	--	--	--	--	--	--	--	--	--	--	--	--	--
	GG + GT + GA	282 (81.3)	182 (81.3)	1 (ref)		162 (82.2)	1 (ref)		20 (74.1)	1 (ref)		51 (83.6)	1 (ref)	
	TT + TA	65 (18.7)	42 (18.7)	1.00 (0.92-1.09)	1.000	35 (17.8)	0.94 (0.60-1.48)	0.780	7 (25.9)	1.52 (0.61-3.76)	0.364	10 (16.4)	0.85 (0.41-1.77)	0.664
*ABCB1* p.Ile1145= rs1045642	CC	83 (24.0)	44 (19.6)	1(ref)		35 (17.8)	1(ref)		9 (33.3)	1(ref)		16 (26.2)	1 (ref)	
	CT	162 (47.0)	122 (54.5)	1.42 (0.92-2.19)	0.113	112 (56.8)	**1.64** (1.03-2.60)	**0.036**	10 (37.1)	0.57 (0.22-1.46)	0.239	26 (42.6)	0.83 (0.42-1.64)	0.596
	TT	100 (29.0)	58 (25.9)	1.09 (0.67-1.78)	0.718	50 (25.4)	1.18 (0.70-2.00)	0.522	8 (29.6)	0.74 (0.27-2.00)	0.549	19 (31.2)	0.98 (0.48-2.04)	0.969
	CT + TT	262 (76.0)	180 (80.4)	1.02 (0.81-1.30)	0.827	162 (82.2)	**1.47** (0.94-2.28)	**0.089**	18 (66.7)	0.63 (0.27-1.46)	0.285	45 (73.8)	0.89 (0.48-1.66)	0.716
*ABCG2* p.Gln141Lys rs2231142	CC	276 (80.2)	191 (86.4)	1 (ref)		167 (85.6)	1 (ref)		24 (92.3)	1 (ref)		56 (87.5)	1 (ref)	
	CA	68 (19.8)	30 (13.6)	**0.64** (0.40-1.02)	**0.059**	28 (14.4)	0.68 (0.42–1.10)	0.116	2 (7.7)	0.34 (0.08–0.47)	0.147	8 (12.5)	0.58 (0.26–1.28)	0.175
*ATM* p.Asp1853Asn rs1801516	GG	254 (75.8)	153 (68.6)	1 (ref)		134 (68.4)	1 (ref)		19 (70.4)	1 (ref)		45 (70.3)	1 (ref)	
	GA	76 (22.7)	64 (28.7)	**1.40** (0.95-2.06)	**0.091**	57 (29.1)	**1.42** (0.95-2.13)	**0.083**	7 (25.9)	1.23 (0.50-3.05)	0.651	15 (23.4)	1.11 (0.59-2.11)	0.740
	AA	5 (1.5)	6 (2.7)	1.99 (0.60-6.66)	0.262	5 (2.5)	1.90 (0.54-6.69)	0.319	1 (3.7)	2.67 (0.39-24.29)	0.380	4 (6.3)	**4.52** (1.16-17.56)	**0.029**
	GA + AA	81 (24.2)	70 (31.4)	**1.43** (0.98-2.09)	**0.061**	62 (31.6)	**1.45** (0.98-2.15)	**0.062**	8 (29.6)	1.32 (0.73-2.40)	0.352	19 (29.7)	1.32 (0.56-3.14)	0.528
*TP53* p.Arg72Pro rs1042522	GG	167 (49.0)	130 (57.8)	1 (ref)		115 (58.1)	1 (ref)		15 (55.6)	1 (ref)		29 (48.3)	1 (ref)	
	GC	150 (44.0)	79 (35.1)	**0.68** (0.47-0.97)	**0.031**	70 (35.3)	**0.68** (0.47-0.98)	**0.039**	9 (33.3)	0.67 (0.28-1.57)	0.355	29 (48.3)	1.11 (0.64-1.95)	0.707
	CC	24 (7.0)	16 (7.1)	0.86 (0.44-1.68)	0.652	13 (6.6)	0.79 (0.39-1.61)	0.511	3 (11.1)	1.39 (0.38-5.16)	0.621	2 (3.3)	0.48 (0.11-2.14)	0.336
	GC + CC	174 (51.0)	95 (42.2)	0.80 (0.61-1.05)	0.104	83 (41.9)	**0.69** (0.49-0.99)	**0.042**	12 (44.4)	0.77 (0.35-1.69)	0.511	31 (51.67)	1.03 (0.59-1.78)	0.927
*ATP7B* p.Ser406Ala rs1801243	TT	103 (30.8)	41 (19.0)	1 (ref)		35 (18.5)	1 (ref)		6 (22.2)	1 (ref)		13 (20.3)	1 (ref)	
	TG	157 (47.0)	113 (52.3)	**1.81** (1.17-2.80)	**0.008**	100 (52.9)	**1.87** (1.18-2.97)	**0.007**	13 (48.2)	1.42 (0.52-3.88)	0.490	32 (50.0)	1.61 (0.81-3.23)	0.174
	GG	74 (22.2)	62 (28.7)	**2.10** (1.28-3.46)	**0.003**	54 (28.6)	**2.15** (1.27-3.62)	**0.004**	8 (29.6)	1.86 (0.61-5.61)	0.270	19 (29.7)	**2.03** (0.94-4.40)	**0.069**
	TG + GG	231 (69.2)	175 (81.0)	**1.90** (1.26-2.88)	**0.002**	154 (81.5)	**1.96** (1.27-3.03)	**0.002**	21 (77.8)	1.56 (0.61-3.99)	0.352	51 (79.7)	**1.75** (0.91-3.36)	**0.093**
*ATP7B* p.Arg952Lys rs732774	AA	103 (30.7)	86 (38.9)	1 (ref)		76 (39.2)	1 (ref)		10 (37.0)	1 (ref)		25 (39.7)	1 (ref)	
	AG	159 (47.5)	96 (43.4)	**0.72** (0.49-1.06)	**0.096**	82 (42.3)	**0.70** (0.47-1.04)	**0.078**	14 (51.9)	0.91 (0.39-2.11)	0.821	31 (49.2)	0.80 (0.45-1.44)	0.471
	GG	73 (21.8)	39 (17.7)	**0.64** (0.39-1.04)	**0.070**	36 (18.5)	0.67 (0.41-1.10)	0.112	3 (11.1)	0.42 (0.11-1.61)	0.203	7 (11.1)	**0.40** (0.16-0.97)	**0.041**
	AG + GG	232 (69.3)	135 (61.1)	**0.70** (0.49-1.00)	**0.047**	118 (60.8)	**0.69** (0.48-1.00)	**0.049**	17 (63.0)	0.75 (0.33-1.71)	0.499	38 (60.3)	0.67 (0.39-1.18)	0.165
*GST* T1/M1 Gene deletions	wt/wt	118 (34.6)	82 (36.8)	1(ref)		72 (36.7)	1(ref)		10 (37.0)	1(ref)		20 (31.3)	1(ref)	
	wt/null	49 (14.4)	18 (8.1)	**0.53** (0.29-0.98)	**0.040**	17 (8.7)	**0.57** (0.30-1.07)	**0.076**	1 (3.7)	0.24 (0.03-1.96)	0.180	6 (9.4)	0.72 (0.27-1.92)	0.512
	null/wt	138 (40.5)	95 (42.6)	0.99 (0.64-1.54)	0.967	82 (41.8)	0.97 (0.65-0.47)	0.90	13 (48.2)	1.11 (0.47-2.64)	0.810	31 (48.4)	1.32 (0.72-2.45)	0.368
	null/null	36 (10.5)	28 (12.5)	1.12 (0.63-1.98)	0.698	25 (12.8)	1.14 (0.63-2.06)	0.667	3 (11.1)	0.98 (0.24-4.04)	0.982	7 (10.9)	1.14 (0.45-2.95)	0.774
*GSTP1* p.Ile105Val rs1695	AA	151 (45.2)	104 (46.4)	1 (ref)		93 (47.2)	1 (ref)		11 (40.7)	1 (ref)		32 (52.4)	1 (ref)	
	AG	162 (48.5)	95 (42.4)	0.85 (0.60-1.22)	0.376	84 (42.6)	0.84 (0.58-1.22)	0.361	11 (40.7)	0.93 (0.39-2.22)	0.873	36 (36.1)	0.64 (0.36-1.15)	0.137
	GG	21 (6.3)	25 (11.2)	**1.73** (0.92-3.26)	**0.090**	20 (10.2)	1.54 (0.79-3.01)	0.199	5 (18.6)	**3.27** (1.03-10.42)	**0.044**	7 (11.5)	1.57 (0.61-4.03)	0.342
	AG + GG	183 (54.8)	120 (53.6)	0.95 (0.68-1.34)	0.776	104 (52.8)	0.92 (0.65-1.32)	0.656	16 (49.3)	1.20 (0.54-2.67)	0.653	43 (47.6)	0.75 (0.43-1.29)	0.296

Statistically significant analyses are in bold.

#### The total group

For the whole group of ovarian cancer patients the polymorphic variants in *PGR, ABCB1, GSTT1, GSTM1* and *GSTP1* genes did not change the risk of developing cancer. Polymorphism p.Gln141Lys in the *ABCG2* gene, borderline significant (OR 0.64; 95% CI 0.40-1.02; p = 0.059), decreased the risk of ovarian cancer for heterozygotes. Polymorphic variant p.Asp1853Asn in the *ATM* gene showed tendency towards increased risk of ovarian cancer for heterozygotes (OR 1.40; 95% CI 0.95-2.06; p = 0.091) and for the carriers of at least one minor A allele (OR 1.43; 95% CI 0.98-2.09; p = 0.061). Significant decrease of ovarian cancer risk was observed for heterozygotes of p.Arg72Pro polymorphic variant in *TP53* gene (OR 0.68; 95% CI 0.47-0.97; p = 0.031). Two studied variants of copper transporter *ATP7B* showed opposite effects on the ovarian cancer risk. p.Ser406Ala polymorphism was strongly connected with the increased risk of ovarian cancer for heterozygotes (OR 1.80; 95% CI 1.17-2.80; p = 0.008), for minor homozygotes (OR 2.10; 95% CI 1.28-3.46; p = 0.003) and for the carriers of at least one minor G allele (OR 1.9; 95% CI 1.26-2.88; p = 0.002). For polymorphic variant p.Arg952Lys in the same gene there was a trend of protective effect against ovarian cancer for heterozygotes and minor homozygotes (OR 0.72; 95% CI 0.49-1.06; p = 0.096 and OR 0.64; 95% CI 0.39-1.04; p = 0.070, respectively). The significant protective effect was shown for the carriers of at least one G allele (OR 0.70; 95% CI 0.49-1.00; p = 0.047). Significant effects were also observed in the analysis of combined *GSTT1* and *GSTM1* genes’ deletion. Haplotype *GSTT1*wt/*GSTM1*null had protective effect against ovarian cancer (OR 0.53; 95% CI 0.29-0.98; p = 0.040) when compared with wt/wt haplotype. Possible increase of ovarian cancer risk was also shown for minor homozygotes of polymorphism p.Ile105Val in the *GSTP1* gene (OR 1.73; 95% CI 0.92-3.26; p = 0.090).

For *BRCA1*- group we observed a significant increase in cancer risk for the heterozygotes carrying silent polymorphism p.Ile1145 = in the *ABCB1* gene (OR 1.64; 95% CI 1.03-2.60; p = 0.036). A less strong effect was observed for the carriers of at least one T allele (OR 1.47; 95% CI 0.94-2.28; p = 0.089). Also, the presence of polymorphism p.Ile105Val in *GSTP1* gene had lost any statistical significance. The rest of polymorphisms under study showed similar effects on cancer risk as observed in the total group.

The *BRCA1*+ group was the only one where p.Val660Leu polymorphism in the *PGR* gene had any impact on cancer risk. This variant significantly increased the risk of developing ovarian cancer for heterozygotes (OR 2.94; 95% CI 1.31-6.58; p = 0.009) and for carriers of at least one minor T allele (OR 2.82; 95% CI 1.28-6.23; p = 0.010). For the trinucleotide polymorphism (rs2032582) in the *ABCB1* gene there was a tendency towards higher ovarian cancer risk for rare TA genotype (OR 5.20; 95%CI 0.87-31.18; p = 0.069). Significantly increased risk of ovarian cancer in this group was also shown for minor p.Ile105Val homozygote in *GSTP1* gene (OR 3.27; 95% CI 1.03-10.42, p = 0.044). It should be noted that the *BRCA1+* group was much smaller than the others and for some rare polymorphic variants the calculations were not possible as there were no subjects carrying the variant under study.

#### Breast and ovarian cancer group

Significant increase of cancer risk was connected with the p.Asp1853Asn polymorphism in the *ATM* gene and minor homozygotes were at higher cancer risk (OR 4.52; 95% CI 1.16-17.56; p = 0.029). Tendency towards increased cancer risk was also found for minor p.Ser406Ala homozygotes in the *ATP7B* gene (OR 2.03; 95% CI 0.94-4.40; p = 0.069) and for carriers of at least one minor allele G (OR 1.75; 95% CI 0.91-3.36; p = 0.093). The second polymorphism of this gene (p.Arg952Lys) was significantly linked with the protective effect against breast and ovarian cancer (OR 0.40; 95% CI 0.16-0.97; p = 0.041) for the minor heterozygotes.

#### Cumulative effects of favorable and unfavorable genotypes on ovarian cancer risk

To examine the cumulative effects of genetic variants under study, regarded as the reflection of gene dosage effect on ovarian cancer risk, analysis of favorable and unfavorable genotypes was performed. This analysis was done for the group of sporadic ovarian cancer patients (*BRCA1*-) to avoid the strong influence of mutated *BRCA1* gene. The polymorphisms, which in univariate analysis were modulating ovarian cancer risk, were included in the groups of risk reductors (favorable genotypes) or risk enhancers (unfavorable genotypes). After stepwise regression analyses, two (p.Arg72Pro in *TP53* and p.Arg952Lys in *ATP7B* gene) and three (p.Ile1145 = in *ABCB1*, p.Asp1853Asn in *ATM* and p.Ser406Ala in *ATP7B* gene) polymorphisms remained in the respective models for each group. The results of analyzing favorable genotypes showed strong significant reduction of ovarian cancer risk in the presence of both variants (OR 0.50; 95% CI 0.30-0.83; p = 0.008). In the analysis of cumulative unfavorable effects we found that ovarian cancer risk progresses in the presence of chosen SNPs, resulting in the highest risk for carriers of all three polymorphisms (OR 7.47; 95% CI 2.08-26.85; p = 0.002) (Table 3).

**Table 3:** **Cumulative effect of favorable and unfavorable genotypes on ovarian cancer risk**

**Analysis**	**Polymorphisms**	**Number of variants**	**Controls n (%)**	**Cases n (%)**	**OR (±95%CI)**	**P**
Favorable effect	p.Arg952Lys *ATP7B*	0	52 (15.7)	46 (23.78)	1 (ref)	
	p.Arg72Pro *TP53*	1	162 (48.8)	96 (49.5)	**0.67** (0.42-1.07)	**0.095**
		2	118 (35.5)	52 (26.8)	**0.50** (0.30-0.83)	**0.008**
Unfavorable effect	p.Ile1145 = *ABCB1*	0	27 (8.1)	3 (1.06)	1 (ref)	
	p.Asp1853Asn *ATM*	1	95 (28.6)	42 (22.3)	**3.98** (1.13-13.97)	**0.030**
	p.Ser406Ala *ATP7B*	2	163 (49.1)	104 (55.3)	**5.74** (1.69-19.50)	**0.005**
		3	47 (14.2)	39 (20.8)	**7.47** (2.08-26.85)	**0.002**

Statistically significant analyses are in bold.

### Survival analysis of SNPs related to cancer risk

**Figure 1:**
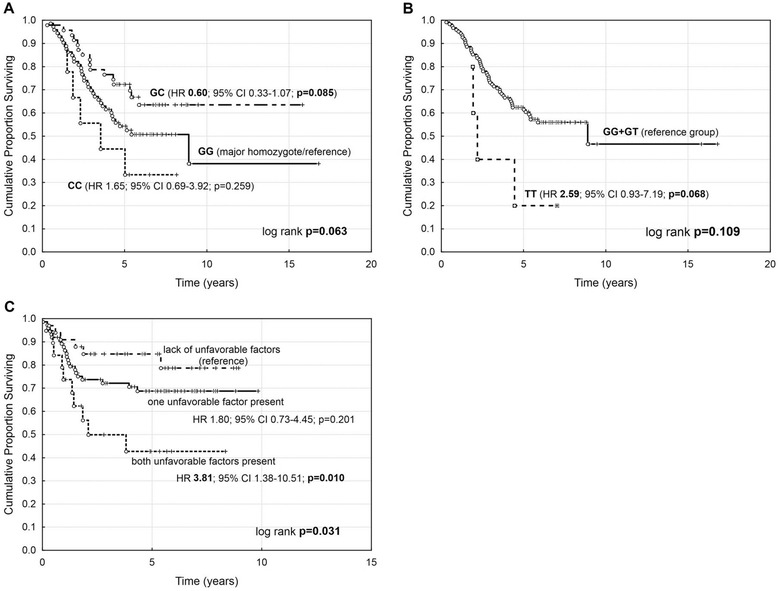
**Survival analysis of ovarian cancer-related SNPs.** **A**. overall survival and risk of death for TP53 p.Arg72Pro; **B**. overall survival and risk of death for PGR p.Val660Leu; **C**. progression-free survival and cumulative risk of progression for unfavorable PFS factors (ATM p.Asp1853Asn GG genotype and ABCB1 p.Ser893Ala/Trp TT + TA genotypes group).

The top SNPs related to cancer risk were subjected to survival analysis for the group of ovarian cancer patients treated with paclitaxel and cisplatin. In the univariate analysis two polymorphisms tended to influence overall survival. Heterozygotes for p.Arg72Pro in the *TP53* gene appeared to have better survival and lower risk of death, when compared to major homozygotes (Figure 1A). An opposite effect was noted for *PGR* p.Val660Leu variant, for which minor homozygotes appeared to cause shorter overall survival and higher risk of death (Figure 1B). However, none of these polymorphisms was a significant prognostic factor after adjustment to FIGO and tumor histotype. As for the progression-free survival, multivariate model adjusted to FIGO and ovarian cancer histotype, we identified two variants as independent factors. Higher risk of disease progression was associated with presence of genotypes lacking a major p.Ser893Ala/Trp polymorphism (HR 2.14; 95% CI 1.07-4.28; p = 0.031) in the G allele of *ABCB1* and with the presence of major homozygote GG of p.Asp1853Asn in the *ATM* gene (HR 2.32; 95% CI 1.06-5.10; p = 0.036) (Table 4). For these high-risk genotypes we performed the analysis of cumulative progression risk in order to calculate the gene dosage effect. The results had shown significant shortening of PFS (Figure 1C) for the carriers of one and both mentioned variants; also, the accumulation of both risk factors was responsible for higher risk of ovarian cancer progression in comparison with persons who did not carry any of the unfavorable genotypes (HR 3.81; 95% CI 1.38-10.51; p = 0.010).

**Table 4:** **Progression-free survival/multivariate analysis/**

**Factor**	**Variant**	**Cases n (%)**	**HR (±95% CI)**	**P**
Histotype	Non-serous	57 (44.2)	1 (ref)	
	Serous	72 (55.8)	**5.04** (2.05-12.37)	**0.0004**
FIGO	1 + 2	58 (46.0)	1 (ref)	
	3 + 4	68 (54.0)	**2.19** (1.03-4.64)	**0.041**
*ABCB1* p.Ser893Ala p.Ser893Thr rs2032582	Common allele carriers (GG + GT + GA)	101 (80.2)	1 (ref)	
	Rare genotypes (AT + TT)	25 (19.8)	**2.14** (1.07-4.28)	**0.031**
*ATM* p.Asp1853Asn rs1801516	Rare allele carriers (GA + AA)	87 (61.0)	1 (ref)	
	Common homozygote (GG)	39 (39.0)	**2.32** (1.06-5.10)	**0.036**

Statistically significant analyses are in bold.

## Discussion

The influence of low-penetrance common genetic variants selected for this study on ovarian cancer risk and survival was not extensively studied yet. Here, in a case–control study we analyzed 12 polymorphic variants and two deletions in nine genes. The case group reflected consecutive cancer cases which included patients with germline mutation in *BRCA* genes and persons who also developed breast cancer. Both groups under study belonged to Slavic ethnic group with specific founder mutations in BRCA1/2 genes [18,19]. Multicenter analyses [20-22] including our own results are done on much larger groups but they often show inconsistent results for the different populations included in the study and in our analysis we wanted to avoid this problem even though the population under study was smaller.

The protein product of progesterone receptor gene (*PGR*) participates in the regulation of epithelial cell proliferation and thus plays an important role in ovarian carcinogenesis. Its expression levels vary several fold in different histological types of ovarian cancer. We wanted to find out whether polymorphic variants of this gene could modulate the risk of ovarian cancer. Previous studies had shown that polymorphic variants of *PGR* were connected with significant increase of ovarian cancer risk (overall [23,24], or limited only to the endometrioid histotype [20]). We chose 2 SNPs (p.Val660Leu and rs474320) from PROGINS allele and one in the promoter region of this gene (rs10895068). In our analysis none of them significantly altered the ovarian cancer risk in the groups under study, except for the *BRCA1*+ group where, for heterozygotes for p.Val660Leu polymorphism, a significant increase of cancer risk was found. This result indicates that p.Val660Leu effect is specific only for carriers of germline mutations in *BRCA* genes, and this finds support in the actual differences between *ER* and *PGR* gene expression in sporadic and hereditary breast cancers, the origin of which has not been elucidated yet. Ovarian cancer is also a hormone-dependent malignancy, so the role of *PGR* gene in *BRCA1*+ and *BRCA1*- ovarian cancers can be different as well. There is also the possibility that the PROGINS variant is co-inherited with the *BRCA1* mutant haplotype [25]. Similar results were also obtained for the rare heterozygotes TA of p.Ser893Ala/Trp polymorphism in *ABCB1* gene. They had a 5-fold higher risk of developing ovarian cancer when compared to the reference homozygotes. *ABCB1* gene product transports a wide range of substrates and it confers resistance to a vast array of clinically and toxicologically relevant compounds. The altered function of *ABCB1* gene product among carriers of rare allele seems to alter the efflux of potentially cancerogenic xenobiotics. Such modification could increase the risk of developing cancer, especially in the group of persons affected by germline mutation in *BRCA1* gene, which affects the DNA repair. Similar associations between the common genetic variants (SNPs) and high-penetrance germline mutations, which additionally modify the risk of developing ovarian cancer, were also found in genome-wide association studies carried out on large cohorts [26]. The authors did not exclude the existence of other modifying risk variants of other genes. Earlier, similar associations with other genes were also found in breast cancer patients also carrying germline mutations in *BRCA1/2* genes [27]. However, analyses done on small groups brought conflicting results. Jakubowska *et al.* found no significant association between the *PGR* gene polymorphisms and the ovarian cancer risk in the group of 146 ovarian cancer patients carrying germline mutation in *BRCA1* gene [28]. Also, in heterozygotes for *ABCG2* polymorphism p.Gln141Lys we found a trend towards decreased risk of ovarian cancer. Similar effects, or no effects at all, were found in other analyses performed for different types of cancer [29,30].

In our analysis a significant increase of cancer risk was observed for *ATM* p.Asp1853Asn heterozygotes which corroborates the finding that missense polypeptides will compete with normal ATM polypeptides in complex formation, and that the functionally abnormal missense polypeptide will sequester key regulators or substrates into nonfunctional complexes, resulting in dominant negative cellular phenotype [31]. Opposite results were found for *TP53* p.Arg72Pro variant. In heterozygotes, we observed a protective effect against tumorigenesis. This means that a lesser number of heterozygotes developed ovarian cancer; moreover, if they were diagnosed with malignancy, their overall survival was better than that of homozygotes. Both effects in *ATM* and *TP53* genes were found only in the total group of patients and the patients without germline mutation in *BRCA1* gene, but not in the *BRCA1+* group. Similar results were also observed by others for breast cancer patients carrying germline mutation in *BRCA1* [32]. It can be concluded that variants under study modify sporadic cancer risk and have no effect on familial ovarian cancer [25,33]. The Arg72 variant exists only in humans and is more efficient in inducing apoptosis and suppressing transformation than the Pro72 allele, which can explain the protective effect found in our analysis [34].

The protective effect of the haplotype *GSTT1*wt/*GSTM1*null was observed for all patients and for the sporadic ovarian carcinoma group. The significant effect for the third glutathione transferase under study, *GSTP1*, was noted only in the familial ovarian cancer group where the carriers of minor GG homozygote were at higher risk of developing cancer. Other researchers did not find any correlations between *GSTT1*, *GSTM1* alone or haplotypes and ovarian cancer risk [35-38]. We observed also the deviation from HWE equilibrium in the control group for the *GSTP1* gene. This effect was observed in this analysis only, so we hypothesize that it is not connected with genotyping errors, population stratification or some other artifacts but rather with the particular gene under study. HWE deviations could arise through important biological mechanisms. In particular, genetic variants having a recessive effect on the successful fertilization and/or development of an embryo might be manifested through such deviations in an unselected sample of “control” subjects [39]. Similar deviation from HWE for *GSTP1* was also noted by others [40].]. Glutathione and its transpherases may regulate the ability of each individual to metabolize environmental carcinogens, which is noticed especially well in smoking-related tumors [41]. Our results show that the genetic variants in glutathione transpherases genes are also able to modify the risk of other cancers which are indirectly related to tobacco use.

Breast and ovarian cancer group shows different behavior from the two groups described earlier. The presence of multiple tumors in one patient is associated with a higher likelihood of genetic susceptibility [42]. In our study, the only significant associations with *ATM* p.Asp1853Asn and *ATP7B* p.Arg952Lys variants indicated the importance of cell cycle and cellular copper metabolism control systems in the cancer-prone phenotype. However, more than one primary malignancy occurs rarely, so our group under study was small and the results of statistical analyses should be taken with caution.

In this study we observed several strong gene-dosage effects. The combined impact of the studied SNPs on ovarian cancer risk shows that ovarian carcinoma can be treated as a polygenic disease partially dependent on the accumulation of unfavorable genetic factors. In our analysis, the unfavorable factors which increased ovarian cancer risk over seven times, prevailed over favorable factors which cut the ovarian cancer risk by half. We can conclude, that carrying unfavorable variants of low-penetrance polymorphisms can increase (OR 7.47 in our study) the ovarian cancer risk to the level similar to the effect of carrying germline mutation in a high-penetrance gene (OR 8.6 calculated for Canadian population [43]). As for the survival analyses, it was evident that accumulation of relatively weak genetic variants (*ABCB1* p.Ser893Ala/Trp and *ATM* p.Asp1853Asn) may significantly enhance the risk of progression after paclitaksel/cisplatin treatment. The trinucleotide polymorphism in the *ABCB1* gene is a widely studied variant linked to altered transporter activity, although the results reported by different authors are inconsistent. It should be noted that in recent large study performed on over four thousand invasive ovarian cancer patients the p.Ser893Ala/Trp polymorphism had no impact on patients’ survival [21]. The *ATM* variant is believed to be responsible for reduced ATM kinase activity and could be the reason for more efficient cisplatin-related apoptosis [31,44]. It could be also the reason why in our analyses the common homozygote of p.Asp1853Asn variant was connected with higher risk of disease progression in the multivariate model.

## Conclusions

Significant associations have been found between the variants of genes involved in: cell cycle regulation, DNA repair, active transport of chemical compounds, detoxification, hormonal regulation of progesterone and the risk of ovarian cancer together with a strong gene-dosage effect. This finding enables to work out the polygenic genetic tests for the non carriers of BRCA1/2 mutation who could have similar risk of developing ovarian cancer as the carriers of germline mutations in BRCA genes if they inherited unfavorable variants.

## Abbreviations

SNP: Single nucleotide polymorphism
OS: Overall survival
PFS: Progression-free survival
OR: Odds ratio
95% CI: 95% confidence interval
HR: Hazard ratio
HWE: Hardy-Weinberg equilibrium
FIGO: International Federation of Gynecology and Obstetrics
RECIST: Response evaluation criteria in solid tumors
MAF: Minor allele frequency
